# *Staphylococcus pettenkoferi*: first report from blood culture in Rio de Janeiro, Brazil

**DOI:** 10.1016/j.nmni.2026.101697

**Published:** 2026-01-09

**Authors:** Bruna Ribeiro Sued-Karam, Julianna Giordano Botelho Olivella, Ana Paula D'Alincourt Carvalho-Assef, Ana Luiza Mattos-Guaraldi, Paula Marcele Afonso Pereira Ribeiro

**Affiliations:** a Laboratório de Bacteriologia Aplicada à Saúde Única e Resistência Antimicrobiana (LabSUR). Instituto Oswaldo Cruz (IOC), Fundação Oswaldo Cruz (Fiocruz), Rio de Janeiro, Brazil; b Faculdade de Ciências Médicas, Universidade do Estado do Rio de Janeiro (FCM/UERJ), Rio de Janeiro, RJ, Brazil

**Keywords:** *Staphylococcus pettenkoferi*, Coagulase-negative staphylococci, Antimicrobial resistance, Biofilm formation, Whole-genome sequencing (WGS)

Dear Editor,

The genus *Staphylococcus* includes more than 70 species of Gram-positive cocci. Coagulase-negative staphylococci (CoNS) are a diverse group of bacteria with variable virulence potential. *Staphylococcus pettenkoferi* is a rare staphylococcal species traditionally associated with bloodstream infections. Although commonly identified as a skin commensal and previously considered clinically insignificant, its role in human infections remains unclear, and its clinical relevance is not fully understood [[1], [2], [3]]. *S. pettenkoferi* was originally isolated and described in 2002, and the only report in Brazil dates from 2010, based on blood cultures from a hospitalized patient in Porto Alegre, Rio Grande do Sul [4].

We describe the first isolation of *S. pettenkoferi* from a human clinical specimen in Rio de Janeiro, Brazil. The organism recovered from the blood culture of a hospitalized patient in Rio de Janeiro in September 2024. Initial identification was performed using MALDI-TOF MS and confirmed by whole-genome sequencing (WGS) on the MiSeq platform and using the CABGen web application and AMRFinderPlus. Antimicrobial susceptibility testing was performed by disc diffusion, and the minimum inhibitory concentration to oxacillin and vancomycin was determined by broth dilution, according to the BrCAST guideline. Biofilm formation on the surface of 96-well polystyrene microtiter plates was performed.

Whole-genome sequencing generated 50 contigs (Fig. 1). Both disk diffusion testing and whole-genome sequencing showed that the isolate was resistant only to tetracycline (*tet(K)_1*). Currently, no standardized multilocus sequence typing (MLST) scheme is available for *S. pettenkoferi,* and no plasmids have been identified. In *Staphylococcus*, biofilm formation is frequently associated with adaptation and persistence, which explains the positive phenotypic biofilm test on an abiotic polystyrene surface. The *arsB* gene was also identified; it encodes an arsenite efflux pump that functions as a uniporter and uses the membrane potential to extrude arsenite and antimonite oxyanions.

**Fig. 1:**
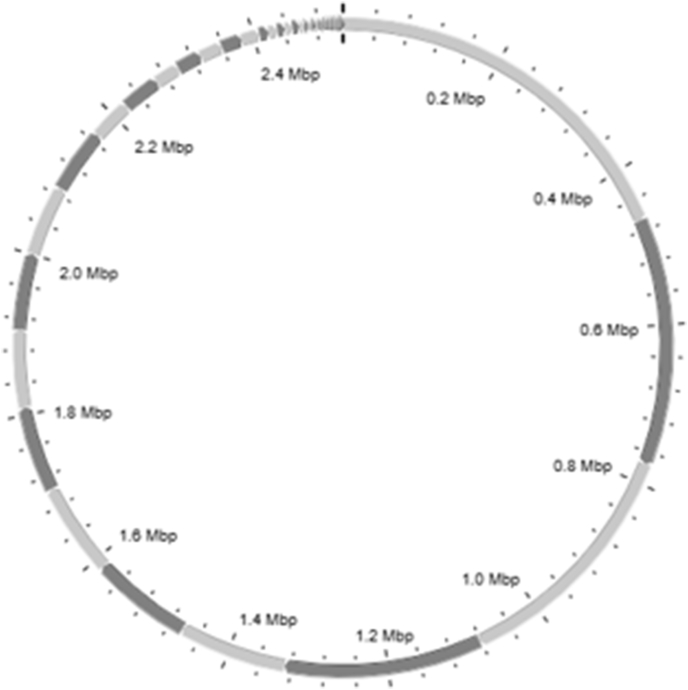
Distribution of contigs generated by whole-genome sequencing of the *Staphylococcus pettenkoferi* strain.

The absence of detectable plasmids suggests that the identified resistance determinant is likely chromosomally encoded or associated with small mobile genetic elements not resolved in the current assembly. This genomic feature may partially explain the isolate's restricted resistance phenotype. The phenotype of biofilm formation may reflect the ability of *S. pettenkoferi* to survive under adverse conditions, such as on medical devices or hospital surfaces. Notably, detection of the *arsB* gene indicates the presence of a heavy-metal resistance mechanism. The presence of this gene suggests environmental or selective pressure exposure and may contribute to bacterial fitness and persistence [5]. Moreover, resistance to heavy metals has been associated with the co-selection of antimicrobial resistance, highlighting the potential ecological and clinical relevance of this finding.

Due to its phenotypic similarity to other coagulase-negative staphylococci, particularly *Staphylococcus auricularis*, misidentification of this uncommon species may occur. In addition, *S. pettenkoferi* has been described as an opportunistic pathogen, complicating the distinction between true bacteraemia and blood culture contamination.

## CRediT authorship contribution statement

**Bruna Ribeiro Sued-Karam:** Conceptualization, Formal analysis, Methodology, Writing – original draft, Writing – review & editing. **Julianna Giordano Botelho Olivella:** Methodology. **Ana Paula D'Alincourt Carvalho-Assef:** Funding acquisition. **Ana Luiza Mattos-Guaraldi:** Writing – review & editing, Funding acquisition. **Paula Marcele Afonso Pereira Ribeiro:** Conceptualization, Formal analysis, Investigation, Writing – original draft, Writing – review & editing.

## Funding

This research was supported by the Fundaç ão de Amparo à Pesquisa do Estado do Rio de Janeiro (FAPERJ; N° E−26/200.538/2025) and the Conselho Nacional de Desenvolvimento Científico e Tecnológico (CNPq; N°. 52/2022).

## Declaration of competing interest

The authors declare that they have no known competing financial interests or personal relationships that could have appeared to influence the work reported in this paper.
